# What Factors Do Allied Health Take Into Account When Making Resource Allocation Decisions?

**DOI:** 10.15171/ijhpm.2017.105

**Published:** 2017-09-12

**Authors:** Haylee Lane, Tamica Sturgess, Kathleen Philip, Donna Markham, Jennifer Martin, Jill Walsh, Wendy Hubbard, Terry Haines

**Affiliations:** ^1^School of Primary & Allied Health Care, Monash University, Frankston, VIC, Australia.; ^2^Workforce Innovation Strategy Education and Research Unit, Monash Health, Clayton, VIC, Australia.; ^3^Department of Health and Human Services, Melbourne, VIC, Australia.; ^4^Monash Health, Clayton, VIC, Australia.; ^5^Centre of Applied Social Research, RMIT University, Melbourne, VIC, Australia.; ^6^State-Wide Equipment Program, Ballarat Health Services, Ballarat, VIC, Australia.

**Keywords:** Resource Allocation, Allied Health, Decision-making, Priority Setting

## Abstract

**Background:** Allied health comprises multiple professional groups including dietetics, medical radiation practitioners, occupational therapists, optometrists and psychologists. Different to medical and nursing, Allied health are often organized in discipline specific departments and allocate budgets within these to provide services to a range of clinical areas. Little is known of how managers of allied health go about allocating these resources, the factors they consider when making these decisions, and the sources of information they rely upon. The purpose of this study was to identify the key factors that allied health consider when making resource allocation decisions and the sources of information they are based upon.

**Methods:** Four forums were conducted each consisting of case studies, a large group discussion and two hypothetical scenarios to elicit data. A thematic content analysis commenced during post-forum discussions of key factors by forum facilitators. These factors were then presented to an expert working party for further discussion and refinement. Transcripts were generated of all data recordings and a detailed thematic analysis was undertaken by one author to ensure coded data matched the initial thematic analysis.

**Results:** Twelve factors affecting the decision-making of allied health managers and clinicians were identified. One of these factors was disendorsed by the expert working party. The 11 remaining factors can be considered to be key decision-making principles that should be consistently applied to resource allocation. These principles were clustered into three overarching themes of readiness, impact and appropriateness.

**Conclusion:** Understanding these principles now means further research can be completed to more effectively integrate research evidence into health policy and service delivery, create partnerships among policy-makers, managers, service providers and researchers, and to provide support to answer difficult questions that policy-makers, managers and service providers face.

## Background

Resource allocation decision-making can take place at all levels within the healthcare system including decisions based on the size of healthcare budgets, which services should be prioritised, what equipment should be purchased and what to offer an individual patient.^1^ Previous authors have described an interplay between the macro-, meso- and micro-level decisions being made, and it is the nature of the decision that dictates who has authority and is best suited to make that decision.^2,3^ Macro-decisions include decisions that affect healthcare needs at a national or state level.^3^ Decisions at this level then inform and influence decisions that affect the healthcare needs at the population level (meso-level). For example when a manager is deciding whether to start a paediatric service or not. These meso-decisions then in turn affect the micro-decisions which include resource allocation decisions that affect the healthcare needs of an individual at the clinical level. Micro level decisions can be separated into service level decision-making or clinical decision-making.^3^ Service level decisions include those made at the department level and clinical decisions include those made at the patient-practitioner level. For example, when a clinician is deciding which patient they should be seeing in their last 30 minutes of a shift. All of these decisions are shaped by the decision-making level that sits above, therefore directly relating to resource allocation.

There are some national bodies that provide guidance as to how healthcare resources should be allocated. In Australia, the National Health and Hospital Reform Commission (NHHRC) have made recommendations regarding specific changes in how healthcare resources should be allocated. This commission identified (among other recommendations) that Australia needs to allocate more resources to prevention and services that are effective earlier in the course of a person’s illness to prevent avoidable hospitalisations.^4^ It was identified that greater amounts should be allocated to provision of subacute services, which are of lower cost per day than acute services. In order to achieve these changes without a relative increase in healthcare funding, it will require reallocation of resources from one sector of the healthcare system to another. This transformation of healthcare expenditure will require many macro, meso and micro-level reallocation decisions to be made. However, without knowing how these decisions are made and the factors that underpin the processes involved, there is no guarantee that the intended transformation will successfully take place.

There are many decision-making algorithms and frameworks that have been constructed to guide clinical decision-making by health professionals. Notable examples include evidence-based medicine, shared decision-making and person-centred care.^5-9^ These frameworks are commonly centred on “bottom-up” decision-making that focus on specific health encounters or conditions, groups of patients, procedures or professional disciplines. A healthcare manager must simultaneously consider multiple health encounters, conditions, patient groups, and professional disciplines, and must therefore take more of a “top down” approach. Thus, there is need to develop a framework that can be used to guide these individuals in making their resource allocation decisions.

One group of health practitioners that can be used to investigate resource allocation decision-making are allied health. Allied health comprises multiple professional groups (eg, dietetics, occupational therapists, physiotherapists, social work) and are organised in a variety of ways. Hospital based services have typically been organised with professional governance within discipline specific departments (eg, speech pathology department) often resulting in competing demands between different clinical specialty areas and the requirements of different funding sources. There is also practical justification for focusing on this group, as they currently administer a sizeable proportion of the healthcare budget.

There are some observations in the literature that might indicate that resource allocation is sub optimal and this brings into question the processes managers of allied health use to allocate resources. For example, in-hospital management of acute exacerbations of chronic obstructive pulmonary disease costs Australia in excess of $550 million annually with significant staffing costs from allied health practitioners.^10^ This is despite little evidence supporting allied health interventions in this setting being available. In comparison, outpatient pulmonary rehabilitation programs led by allied health professionals are provided to less than 10% of people who would benefit, yet could reduce disease-related hospitalisations by up to 75%.^11^ This problem is likely to be broader than this one clinical area as studies from the Netherlands, Australia and United States suggest that at least 30%-47% of patients do not receive care consistent with current scientific evidence.^12,13^ Thus there is need to understand how managers of allied health are making their resource allocation decisions.

This research aims to identify the factors that allied health managers consider when making resource allocation decisions, those factors that they think should be considered but currently are not, any that they think are considered but should not be, and the sources of information they rely upon for their decisions. From this, we sought to define and standardise a set of decision-making principles that can be used to underpin resource allocation decisions in clinical practice and allied health service planning.

## Methods

### Design

This was a qualitative study that used an ethnography design to focus on a ‘detailed and accurate’ description rather than explanation.^14^ In particular the study was interested in understanding the factors decision-makers consider rather than abstract interpretations.

### Participants and Settings

The study was interested in the perspectives of the mangers of allied health making resource allocation decisions. Participants included managers of allied health and allied health clinicians, managers, academics and policy-makers working in a range of settings in regional and metropolitan health services in Victoria, Australia. For example, nurses could be included in this study if they were the managers of allied health practitioners. No minimum amount of clinical/managerial experience was required. Managers of allied health are responsible for departments of varying size, typically made up of 1-2 disciplines. For example, dietetics and speech pathology. Many public hospitals in Victoria provide allied health services to co-located private hospitals. The decision-making approval mechanisms are inherently different between the two sectors, yet it can be the same people who make allied health resource allocation decisions for both contexts. Although most of our participants worked in public health settings, some may have had responsibility for providing allied health services to co-located private facilities. The study used a convenience sample but included a changing location strategy across one regional, one inner metropolitan and one outer metropolitan area so that the four forums of up to 20 participants each would draw a range of stakeholders from different health services across the state of Victoria.

Fifty-nine managers of allied health and clinicians were interviewed. Our sample was predominantly female with heavy representation from the physiotherapy and occupational therapy professions (Table).

**Table:** Participant Demographic Data

**Characteristic**	**Total (n = 59)**
Gender	
Female	45 (76%)
Age	
20-29 years	1 (2%)
30-39 years	14 (24%)
40-49 years	24 (41%)
50-59 years	18 (30%)
60+	2 (3%)
Discipline background	
Audiology	3 (5%)
Dietetics	6 (10%)
Exercise physiology	1 (2%)
Nursing	1 (2%)
Occupational therapy	11 (19%)
Physiotherapy	19 (32%)
Podiatry	4 (7%)
Prosthetics and orthotics	1 (2%)
Psychology	2 (3%)
Social work	3 (5%)
Speech pathology	6 (10%)
Did not record	2 (3%)
Years of allied health clinical experience [mean (SD), range]	18 (0.39), 0-40
Years of allied health managerial experience [mean (SD), range]	9 (0.38), 0-32
Participants with research experience	8 (14%)
Participants with teaching experience	7 (12%)

An expert working party was also formed consisting of a consumer representative, two investigators, and nine allied health managers (drawn from the group interviews) to aid in the process of member checking during data analysis. Our sampling approach was designed such that we anticipated it would be sufficient to reach saturation in addressing our research aims across the four meetings.^15^

### Procedure

Participants were recruited via email advertising through the Victorian Allied Health leadership Council circulation list including practitioners from both clinical and academic backgrounds. Local recruitment was also completed at Monash Health, Victoria, Australia. Snowball sampling whereby managers were asked to identify additional colleagues who may be interested in participating was also adopted. Potential participants were sent information regarding the study procedures prior to study forums taking place, which included instruction on how to prepare their real-life case study. Participants were informed that their attendance at the forum was taken as implied consent for their involvement in this study and that their responses were being audio recorded. Each forum followed the same semi-structured format and was conducted by the same project investigators to ensure consistency in the techniques being applied. The forums took place in August and September 2014. Each participant attended one forum, each of which was 2.5 hours in length. The forums were digitally recorded and later transcribed verbatim.

### Measurement

The forums followed a semi-structured format to allow for flexibility in the order of topics covered and to allow specific issues to be explored in further detail. Participants were told to prepare a case study prior to the group meeting describing how they recently made a resource allocation decision. They were asked to describe the barriers/facilitators they encountered in implementing these decisions. These case studies were each discussed, one at a time, in a small groups (3-4 participants) facilitated by one of the authors or one of the project steering committee members. Participants were then asked in a large group (10-19 participants) to describe, in an ideal situation, the factors they thought should be taken into account when making resource allocation decisions and identify why there were differences between the “ideal” situation and the current reality. Hypothetical scenarios were then worked to explore if there were differences in how decisions were made depending on whether the decision involves provision of additional resources or reallocation of existing resources (taking from one area to give to another). These were completed in the same small groups as the case studies. The groups were presented with one of the following hypothetical scenarios:

(A) You are responsible for providing an Allied Health Community Health Service for a geographical area and there is a sudden increase in refugee need with no increase in resources.

(B) You have a new Chief Executive who is passionate about paediatrics and has allocated an additional sum of money to Allied Health for use within the paediatric program. Within your health service, paediatrics comprises the emergency department (ED), neonatal intensive care unit (NICU) and special care nursery (SCN), paediatric intensive care unit (PICU), acute wards, rehabilitation and community services.

Participants were then asked the following questions; (*i*) How would you decide where to take resource from in order to allocate additional resources? (*ii*) What information would you need? (*iii*) Where would you go to get it?

### Data Analysis

Data collected from the forums were analysed using directed content analysis.^16^ This approach was adopted as it supports the use of existing research and theory to gain an increased understanding of prior conceptualisations and the development of new understandings. Data analysis involved key word recording/searching and thematic analysis of all forum transcripts. Key emergent factors were identified during post-forum discussions by forum facilitators. The expert working party were then presented with the key factors generated from the forums, to expand on/critique any factors they felt were not adequately captured in the data. This was used as a process of member checking to aid trustworthiness of data interpretation.^17^ A detailed thematic content analysis was then undertaken by one author (HL). Transcribed audio files were coded using QSR NVIVO 11 software. Coded data were matched onto the emergent factors previously identified. The list of emergent factors were checked for completeness against the coded data to ensure there were no further factors emergent that had not already been identified during the post-forum discussions or through the expert working party member checking process. These factors included extent of the problem, effectiveness, cost effectiveness, access, reputation, interdependent services, workforce, internal considerations, external considerations, stakeholder support, resourcing and equity between disciplines. The process described by Potter and Levine-Donnerstein^18^ was used to initially define these key factors and then identify text in the transcripts that contained content that aligned with these key factors. The transcript data provided more specific information on these pre-determined factors and they were clustered into three thematic areas of Appropriateness, Impact and Readiness. For instance the key factors of stakeholder support and resourcing were clustered together in the Readiness theme. There were no new factors identified in the transcripts.

## Results

Data analyses identified several factors relevant to our research aims from each forum, though no new factors were identified in the third and fourth forum that had not already been identified in the first two forums. Twelve factors affecting the decision-making of allied health managers and clinicians were identified. These factors were drawn from the case studies (real life decision-making) and large group discussion (ideal decision-making). By the end of the forth forum, factors identified were found to be overlapping in both the real and ideal data collection areas. One of these factors was disendorsed by the expert working party as they felt that although this was identified as a factor that currently influenced decision-making, that it should not be taken into account when making resource allocation decisions. The remaining factors identified were endorsed by the expert working party and can be considered to be key decision-making principles that should be consistently applied in allied health resource allocation decisions. These principles were clustered into three overarching themes (Figure) and will now be discussed individually within each thematic area.

**Figure:**
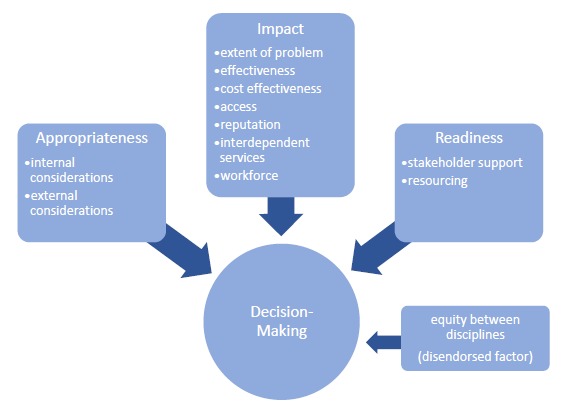
Key Principles Identified.

### Appropriateness

Principles in the “Appropriateness” theme describe the context of the broader change and relate to the consistency the option has with organisational objectives and broader policy directions.

### Internal Considerations

Participants reported that the decision-maker needs to consider the strategic direction and goals of the organization, key targets and performance indicators, the risks for the organization and how the proposed change aligns with these.

*“If we were doing it within [a health service] I’d be going, ‘Is this on [health service’s] strategic services clinical services plan?’”* (Participant, hypothetical scenario, forum three).

Participants also reported the proposed change needed to be consistent with the organization’s core business and address a service gap,

*“…Yes it might increase health and it might be cost effective but that doesn’t necessarily mean that my service should be the one providing that service.”* (Participant, hypothetical scenario, forum three).

It was also found that the decision-maker should also consider collaboration both internally and externally. Background contextual principles that may impact on making the decision to change at that particular point in time was also reported in terms of the timing of the decision.

*“So each one of us would have had a history where if I look at the fairly major changes that have impacted on staff in the last 12 months, that is accelerating over time, have a bit of change fatigue in Allied Health I think.”* (Participant, case study, forum two).

There was recognition that internal considerations may impact both on whether a decision to change is made or when the change is to be implemented. These considerations included change fatigue (other recent changes within an organisation that make staff less capable or inclined to change further in the short term), personnel changes (this may both enable or hinder broader structural changes to take place), impending policy changes (governments may flag initiation or closure of programs/activities which may both enable or retard other changes from being made), and other environmental changes (moving sites, merging wards and other changes to the service environment may both enable or retard changes from being made).

*“We’d always wanted to do something different, but we’d had a couple of long term people in jobs that would not have been happy to make the kind of change that we wanted to make. So we used this little moment when we had a couple of staff turnover to essentially introduce a whole lot of change”* (Participant, case study, forum four).

### External Considerations

External considerations included what the experts in the field and/or government bodies or professional bodies are recommending. Participants stated government policy and recommendations could also dictate that type of services you provide.

*“We talked about needing to make sure we knew what policy directions there were, like the outside of our area, sitting under the Department of Health and that kind of thing, because that might confound some of the initiatives if they were going in the wrong direction”* (Participant, hypothetical scenario, forum four).

Considerations of services provided by other accessible organisations were also discussed,

*“If there were existing agencies that might not be part of your own, but are still providing a service, then you wouldn’t double up on that service”* (Participant, group discussion, forum three).

### Impact

Principles in the “Impact” theme relate to the change that the proposed option may have on effective and efficient health service delivery and on the healthcare service.

#### Extent of the Problem

The extent of the problem was reported to be the perceived demand/need for the proposed change by the decision-maker including the present and future anticipated burden and the frequency and severity of the problem in the population of interest. They discussed the notion that problems that had higher levels of frequency and burden should potentially be prioritised in resource allocation decision-making. The extent of the problem for the decision-maker was also influenced by the services that were already provided at a local level to address that problem.

*“So I would want to understand what the health needs are to then determine how that growth could potentially map across the services I’ve got, and what that would mean in terms of the demands for those services; and I’ll make it up, but say there’s a trend in psychiatric services and diabetes, and then I would be looking at how I can bolster those services to meet those needs”* (Participant, hypothetical scenario, forum four).

Extent of the problem also highlighted the need to consider the long-term health benefits from the change including the preventative considerations both short and long term of the proposed change. Several participants identified that change can bring about both positive and negative impacts, necessitating that overall benefit to the community be considered.

*“What’s the bigger picture here? What are we actually trying to achieve as opposed to just addressing the dripping tap?”* (Participant discussion, hypothetical scenario, forum two).

#### Effectiveness

Participants reported they obtained evidence of the effectiveness of the proposed change from multiple sources including sourcing information from local data originating within the healthcare organisation and external data such as published literature.

*“So to give you quick social work example, if I look at the literature and the literature says the most effective family meetings should be an hour, let’s say how I look at my data that’s very accurate and half my staff chair family meetings over two hours, that just helps me, informs me to have those discussions with people about how we are using our resource and the change of process, I guess with staff”* (Participant, group discussion, forum one).

It was identified that in an ideal world, published data would assist in making all decisions however participants found often it was harder to access or apply research directly to their specific situation.

*“I think sometimes literature is helpful there but you have to know where to find it”* (Participant, group discussion, forum two).

Participants also reported identifying what other “like” services are doing to address the issue,

*“This is what our service may look like in terms of money. Who else has a similar sized service? And talk to them about their demand and where their experience of - how they’ve structured their service”* (Participant discussion, hypothetical scenario, Forum one).

Participants reported benchmarking as a way of determining if the strategies like services were using were having good outcomes and could use evidence of this as a way to determine if this was appropriate for their organisation/service. This process is about learning and further adding to the current ideas to create better outcomes rather than copying other like services.

*“Benchmarking. …See what their ideal would be and then what they have learnt...The way they’re structured. What they see is a real gap with how they’ve done it. So we could brainstorm it or maybe troubleshoot us not making the same mistakes”* (Participant discussion, hypothetical scenario, forum one).

#### Cost-Effectiveness

Participants reported considering if the change was going to be economically efficient relative to alternate ways of using the same resource.

*“The other thing I’m thinking is let’s just say it’s X amount. Well X amount in the NICU may not go as far as it would in ED, for example. So it’s a bit of that cost effectiveness thing in terms of where we’re going to get the best outcome for the best dollar”* (Participant discussion, hypothetical scenario, forum three).

#### Access

Impact on access to health services was reported to focus on the specific health needs and enable referral to other health services in the region to address other issues that impact on health outcomes (eg, transport, financial counselling, housing). This also included considering greater health outcomes for consumers and greater reach to more consumers.

*“But it’s also health equity for the consumer… That Jimmy that lives in [Regional location] and has to go and see [health professional], can get the same service as Suzanne who can see me in [Metropolitan location]”* (Participant, group discussion, forum three).

#### Reputation

Participants considered the risk of a potential change within an organisation. Reputation included direct risks to patients and health outcomes as well as risk of negative media influences and the affect this has on public perceptions.

*“I would also start thinking about risk… Reputational risk, patient risks so severity, likelihood of something going very, very wrong and landing my head honcho person in the papers anyway”* (Participant, hypothetical discussion, forum three).

#### Interdependent Services

Another consideration was that if one area of service provision is modified, this could lead to positive and/or negative impacts on other services.

*“If you increase funding to the NICU [neonatal intensive care unit] or into the PICU [Paediatric Intensive Care Unit], or whatever, what’s going to be the impact on where those kids are going? Yeah, the flow on effect”* (Participant discussion, hypothetical scenario, forum three).

#### Workforce

The likely impact of the proposed change would have on the workforce was also reported. For example, if discontinuing a service, loosing a staff member with key skills may impact the health service as a whole. It also included services changes that reduce the workforce’s capacity or enhances it.

*“That it’s, again, that sort of universal principle which is that you recognise the need for specialty, but at the same time it might be better to flex people across the system. So you have to meet specialty with the right kind of skillset, but at the same time provide the ability to have some more generic group of workers who can flex with those systems”* (Participant, case study, forum one).

### Readiness

Principles in the “Readiness” theme relate to the ability of the health service to provide the proposed option.

#### Stakeholder Support

The readiness for the proposed change by relevant stakeholders was reported in individual case studies and large group discussions. Participants felt changes were easier to implement if there was existing acceptance.

*“So I guess they were on board. They were ready. They saw that there was issues and that things needed to change, so that made the change management a lot easier”* (Participant, case study, forum one).

The stakeholder support principle included local lobbying and feedback from staff or other stakeholders internal or external to the organization. This feedback may be based on perceived impacts on patient care effectiveness or impact on economic efficiency, workload, and job satisfaction. Some recognized this as a barrier to implementation more than a principle that influenced decision-making, although others raised examples where it did influence their decision-making.

*“So we actually got to a spot where we just needed to just stop for a bit. It was just decided that we’re just not getting anywhere”* (Participant, case study, forum one).

Consumer perspectives of the proposed change and the value of consumer contributions were important considerations in stakeholder support.

*“I think if we focus everything back to the consumer and the patient then that’s the way that we need to look at how do we change our resources or utilise our resources and asking the consumer ‘Where do you want these resources?’”* (Participant, case study, forum three).

#### Resourcing

Resourcing described the practical considerations and barriers that would prevent the change proceeding within an organization, at least in the short term. Including whether the organization has access to staff with the appropriate skill set, access to equipment that staff use to support their practice and access to capital including buildings, rooms, and other items that are needed for the health service to function.

*“Do we have the staff available to meet the roles… no point trying to create a role for something where you know you’re not going to be able to recruit into”* (Participant, hypothetical scenario, forum one).

Participants described that the amount of resource available often also defines the scope of how large the proposed change can be.

*“We would then need to think about the greatest need, the greatest risk, I guess, for social work. So for us, we might be thinking more about the high-risk sort of child protection cases if I had 10 EFT. Whereas if I had 35, I could still do that, but then I could also look more broadly. So it depends on how many people I can put on”* (Participant, hypothetical scenario, forum one).

Sustainability of the resources and funding was also important to consider. It was reported that funding rules and restrictions often guide what changes can occur, and that changes that are initially implemented with “project” staff without ongoing funding often fail to be sustainable once project funding concludes.

*“…needing to make sure that the funding was sustainable, making sure that it was recurrent and that we would be able to continue”* (Participant, hypothetical scenario, forum four).

How resources are currently being utilized at a local level was also considered including the potential to modify an existing service,

*“We’re going through a change impact process right now in a particular program where we’re closing down a team and moving the EFT to an area where we think there’ll be more bang for its buck”* (Participant, case study, forum one).

This can be done by identification and exploration of alternative potential changes that could be considered including alternative models of care, diversity of the workforce and considering any other innovative changes.

*“You’d come up with two or three options where you thought, I could put it there, I could put it there, I could put it there… Which option would we maintain the best options meeting the needs for the whole community and which one is the most sustainable”* (Participants, hypothetical scenario, forum three).

#### Disendorsed Factors

##### Fairness Between Disciplines

The concept of the change to be proportional in how different subgroups/disciplines within an organization will be affected by a decision was also discussed. This included managing the perceptions of “unfairness” by staff and maintaining existing hierarchies in total funding allocations between disciplines/subgroups.

*“I guess the other key driver was we’ve tackled it from an equity perspective in terms of the percentage of the budget that people have and then making the percentage of the savings. My issue with that is that it’s based on historical allocations, not an actual needs base thing so that was a big toss up”* (Participant, case study, forum two).

The expert working party agreed equity between disciplines should not guide resource allocation decisions due to focusing on the professional practice rather than considering patient outcomes.

*“We should be looking at a distribution that’s equally available to patients or patient groups rather than our own professional background”* (Participant, expert working party).

##### Sources of Information

Interwoven in the data describing the considerations managers have when making their resource allocation decisions were data describing the types of information they relied upon to make their decisions and where they sourced this information. The results have highlighted participants followed three different pathways of obtaining evidence for making resource allocation decisions; External sources of information (exploring published literature), contacting similar services for sources of information or relying on local sources of information. Managers and clinicians reported seeking and using information that was quick and easy to obtain, and easily understood. It was evident that evidence obtained to guide decisions was mostly sought from local data or from similar services, and that seeking and using information directly from the published literature was rare. Many expressed desire to use evidence but had difficulty identifying, understanding, synthesising, and using it to answer the specific question they were faced with. The ill fit between the decisions they had to make and the related research evidence was also discussed,

*“The guidelines or best practice sort of stuff may or may not exist, in some areas it certainly does. Does it go down to the nitty gritty of you know EFT allocations of disciplines per, not in many instances, but you can certainly use some of the you know, like the NICE guidelines to help frame your – the model that you want to implement”* (Participant, hypothetical scenario, forum three).

Others expressed concern about how much time and resources it took to use the evidence base to inform their decisions,

*“Well I think that’s a factor but from my point of view there isn’t really good data that says this given group, this intervention provides this outcome most cases... Well we don’t know how to access it… I think it’s not easy to find”* (Participants, group discussion, forum two).

This often resulted in managers using other sources to inform their decisions, where they would most often ask other services what they did to address a similar problem.

## Discussion

This study has identified several principles of decision-making that currently influence how managers of allied health make their resource allocation decisions. It should be noted, particularly with regard to the case studies that were collected, that these principles were not universally considered.

The principles identified in this study are consistent with previous literature exploring key concepts of resource allocation decisions in allied health.^19-22^ A recent narrative review by Angelis et al identified several different priority systems for broader health service resource allocation decision-making.^23^ Mapping these against the principles identified in this study illustrate that the findings are broadly consistent with pervious literature although there are additional factors that were not identified in the present study. For example, in the Netherlands, in 1990, the Committee on Choices in Health Care endorsed a set of four priority principles: necessity, effectiveness, efficiency and individual responsibility.^24^ Necessity, effectiveness, efficiency are conceptually comparable to the principles of effectiveness, cost effectiveness and extent of the problem that were identified in the present study. Individual responsibility was not identified as factor in the present study and was based on if it was acceptable for the individual to pay for services themselves.^23^

In Sweden in 1992 the Parliamentary Priorities Commission identified that priority setting should follow a set of three core principles including human dignity, need and solidarity, and cost-efficiency.^25^ These latter two principles correspond with the principles of extent of the problem and cost effectiveness identified from the present study respectively. Human dignity was described as the view that all people are equal.^26^ It is not clear what the ramifications would be if this principle had of been identified in the present study. One could argue that this principle maybe be at odds with the positive discrimination policies in some healthcare settings around the world which preferentially target certain groups with healthcare resources in order to address health disparities.

The literature supports the principles identified however there is little evidence that illustrates how these principles are weighted, how they influence decisions, and how to guide decision-making in regards to service priorities of a healthcare system as a whole, with much of the evidence that does exist being out of date.^27^ The principles identified appear to be interconnected and interdependent, with most being difficult to use in isolation. The range of principles demonstrates the complex decisions decision-makers are faced with when considering the competing demands of a healthcare service. It is essential decision-makers can follow a consistent approach and can provide justification for their decisions.^28^ As previously discussed, resource allocation decision-making often is also based on historical allocations.^29,30^ This can be problematic as the needs of consumers are constantly changing, and following historical allocations of resources may no longer be meeting the current needs of the population. Successful resource allocation decision-making is a desirable goal for managers however due to the lack of explicit guidelines, there is no way of knowing if an organisation has gone down the correct path to achieve best outcomes.^20,31^ Some authors have voiced opinion that this kind of decision-making process is associated with considerable uncertainties and has been more about ‘muddling through’ rather than explicitly using a process in which the sciences have had an influential part to play.^21,32^ This indicates that consistent decision-making processes may require support from a framework to facilitate consistent application of these principles in real-life decision-making.

This study also identified that use of research evidence in these decisions was limited, that participants identified having limited capacity to do this and that they would benefit from having others address these problems so that they could rapidly use pre-processed information to inform their decisions. Previous literature reports common barriers of using literature or publications by allied health as including too much scientific information, lack of clinical information, lack of generalizability of the literature to a specific client population, difficulty interpreting results and lack of time.^33,35^ In the present study, participants identified several barriers they faced that were consistent with these. Specifically they reported that it takes too much time, they had limited capacity and that the research question that is answered is not the same as the management decision that they are confronted with. For example, the evidence may suggest attending a falls and balance clinic may reduce the risk of falls in elderly people however this does not inform the manager how many allied health professionals they need to employ in order to run this service effectively. Even when there is common agreement between researchers about the causes of a particular health problem and effective treatments for their management, the ambiguity in research outcomes, questions about the validity of generalising results to different populations, cost effectiveness judgments and politics, make it extremely difficult to identify a single best solution of allocating scarce resources.^21,36^ Hence, although managers rarely rely on research evidence to inform their resource allocation decisions the paucity of evidence generated to address the questions that they face makes it difficult for them to do so.

Participants may have been reluctant to talk about failures when in open discussion with peers throughout the forums. This may have resulted in missing factors or principles based on case studies. Due to this concern, hypothetical scenarios and group discussions were also included in the forums. In the latter, people could talk about the failed decisions they have seen others make. The expert working party were used to confirm initial findings and were given opportunity to discuss any considerations they felt were missing from the data. This was done to try to avoid missing important principles that should be when making resource allocation decisions. It is possible the factors identified were influenced by an order effect due to the data collection approach where each forum commenced with the real life case studies before progressing to the ideal situation. Participants who took part in the forums included Victoria allied health managers and clinicians. Decision-making principles may differ in other states or countries due to the differences in relationships and processes between governments and health authorities/managers.

There are several policy recommendations that can arise from the present study. First, although the study identified a broad range of principles, it was also identified that within each case study only a smaller subset was discussed. This means the current decision-making processes are likely to be incomplete and that an appropriate policy response would be to develop tools to assist decision-makers to consistently apply the complete set of principles identified in this research. Such a tool if delivered from an online medium could be used to collect data and be used to monitor principles that were more influential on final decisions being made. This would allow policy-makers to know whether resources are being allocated in a manner consistent with their local public policy settings.

Future research is warranted to determine if the decision-making principles identified amongst allied health staff are consistent with those applied by medical and nursing staff and generic hospital administrators. It is also warranted to investigate a means to enhance allied health managers using research evidence as a source of information to underpin their decisions. Studies could potentially investigate whether using evidence, pre-processed by a team of academics, managers, clinicians and consumers, could assist managers to better use research evidence to inform their decision-making.

## Acknowledgements

Funding for this project was provided by the Victorian Department of Health and Human Services. The authors would like to thank study participants for contributing their time and insights to this project. TH was supported by an NMHRC Career Development Fellowship (APP1069758).

## Ethical issues

This project received approval from the Monash Health Human Research Ethics Committee (14293Q).

## Competing interests

Authors declare that they have no competing interests.

## Authors’ contributions

HL was a member of the project steering committee, facilitated the data collection forums, participated in the preliminary data analysis post forum discussions, completed the detailed thematic analysis of the data and drafted and completed final write up of the manuscript. TS was a member of the project steering committee and project manager, facilitated the data collection forums, participated in the preliminary data analysis post forum discussions and assisted with final write up of manuscript. KP was a member of the project steering committee, facilitated the data collection forums, participated in the preliminary data analysis post forum discussions and assisted with final write up of manuscript. DM formulated the idea for the project, was a member of the project steering committee, facilitated the data collection forums, participated in preliminary data analysis post forum discussions and assisted with the final write up of manuscript. JM assisted with the detailed thematic analysis of the data and assisted with the drafting and final write up of manuscript. JW was a member of the project steering committee, facilitated the data collection forums, participated in the preliminary data analysis post forum discussions and assisted with final write up of manuscript. WH was a member of the project steering committee, facilitated the data collection forums, participated in preliminary data analysis post forum discussions and assisted with final write up of manuscript. TH formulated the idea for the project, was a member of the project steering committee, facilitated the data collection forums, participated in the preliminary data analysis post forum discussions and assisted with the drafting and final write up of manuscript.

## Authors’ affiliations

^1^School of Primary & Allied Health Care, Monash University, Frankston, VIC, Australia. ^2^Workforce Innovation Strategy Education and Research Unit, Monash Health, Clayton, VIC, Australia. ^3^Department of Health and Human Services, Melbourne, VIC, Australia. ^4^Monash Health, Clayton, VIC, Australia. ^5^Centre of Applied Social Research, RMIT University, Melbourne, VIC, Australia. ^6^State-Wide Equipment Program, Ballarat Health Services, Ballarat, VIC, Australia.

## Key messages

**Implications for policy makers**

- Factors identified and endorsed by the expert working party can be considered to be key decision-making principles that should be consistently applied in allied health resource allocation decisions.
- Participants agreed it would be useful to use these principles in decision-making such that they formed a decision-making framework/tool.
- A decision-making tool could be used to monitor principles that were more or less influential on final decisions made.
- This would allow policy-makers to know whether resources are being allocated in a manner consistent with public policy settings.

**Implications for the public**

This research has identified key decision-making principles used by allied health managers when making resource allocation decisions. Future research can now be completed to identify a framework or tool that can facilitate consistent application of these principles in real-life decision-making including allied health clinical practice and service planning.
